# Unveiling cryptic phenology and environmental responses in subtropical evergreen broad-leaved tree canopies using phenology camera observations

**DOI:** 10.3389/fpls.2026.1830218

**Published:** 2026-07-06

**Authors:** Lu Yang, Xiang Niu, Bing Wang, Tingyu Xu, Qingfeng Song, Fengshi Pan, Keda Wang, Yucheng Wang, Xiang Ma

**Affiliations:** 1 College of Forestry, Shenyang Agricultural University, Shenyang, China; 2 Ecology and Nature Conservation Institute, Chinese Academy of Forestry, Beijing, China; 3 Dagangshan National Key Field Observation and Research Station for Forest Ecosystem, Xinyu, China; 4 Key Laboratory of National Forestry and Grassland Administration on Forest Ecosystem Conservation and Restoration, Beijing, China; 5 SINTEF Industry, Oslo, Norway

**Keywords:** Cryptic phenology, Evergreen broad‑leaved forest, Subtropical China, Phenology camera, Canopy vegetation index, Red-Green Vegetation Index, Double logistic model, Environmental lag effects

## Abstract

**Introduction:**

Seasonal canopy changes in subtropical evergreen broad-leaved forests are subtle, making it challenging to quantify phenological dynamics and their climatic drivers.

**Methods:**

Using phenology camera (*PhenoCam*) imagery from evergreen broad-leaved trees in Jiangxi Province, China, collected in 2024, together with environmental variables spanning 2023 and 2024, four vegetation indices - the Normalized Difference Vegetation Index (*NDVI*) and RGB-based chromatic indices, including Green Chromatic Coordinate (*G_cc_*), Red Chromatic Coordinate (*R_cc_*), and the derived Red-Green Vegetation Index (*RGVI*) - were extracted to evaluate their performances in resolving phenophases and to identify environmental controls. Multiple meteorological variables were reduced to a small set of independent climatic variables using principal component analysis, and related to latent phenological indicators via Pearson correlation analysis. Phenological transition dates were estimated by fitting a double Logistic model to the time series data, after which the effects of lagged environmental variables on each transition were quantified.

**Results:**

The analysis shows that *R_cc_* and *RGVI* most closely tracked seasonal climate variation and outperformed *NDVI* and *G_cc_* for phenophases detection. Canopy dynamics were primarily associated with radiation, air temperature, moisture availability and atmospheric pressure. Based on *RGVI* double logistic fitting, the growing season commenced in early April (day-of-year (*DOY*) 98), peaked by late April (*DOY* 116) and commenced a significant decline in early December (*DOY* 340), spanning a growing-season length of 242 days. The timing of phenological events showed clear carry-over effects: prior-season environmental anomalies exerted lagged influences on subsequent canopy development, after certain threshold conditions were exceeded.

**Discussion:**

Overall, our findings clarify that near-ground *PhenoCam*s provide sensitive, scalable indicators of evergreen canopy dynamics in the subtropics. Red-Green Vegetation Index offers reliable phenophase detection, and incorporating cross-season lag effects will improve the understanding of phenological mechanisms in evergreen ecosystems.

## Graphical Abstract

**Figure:**
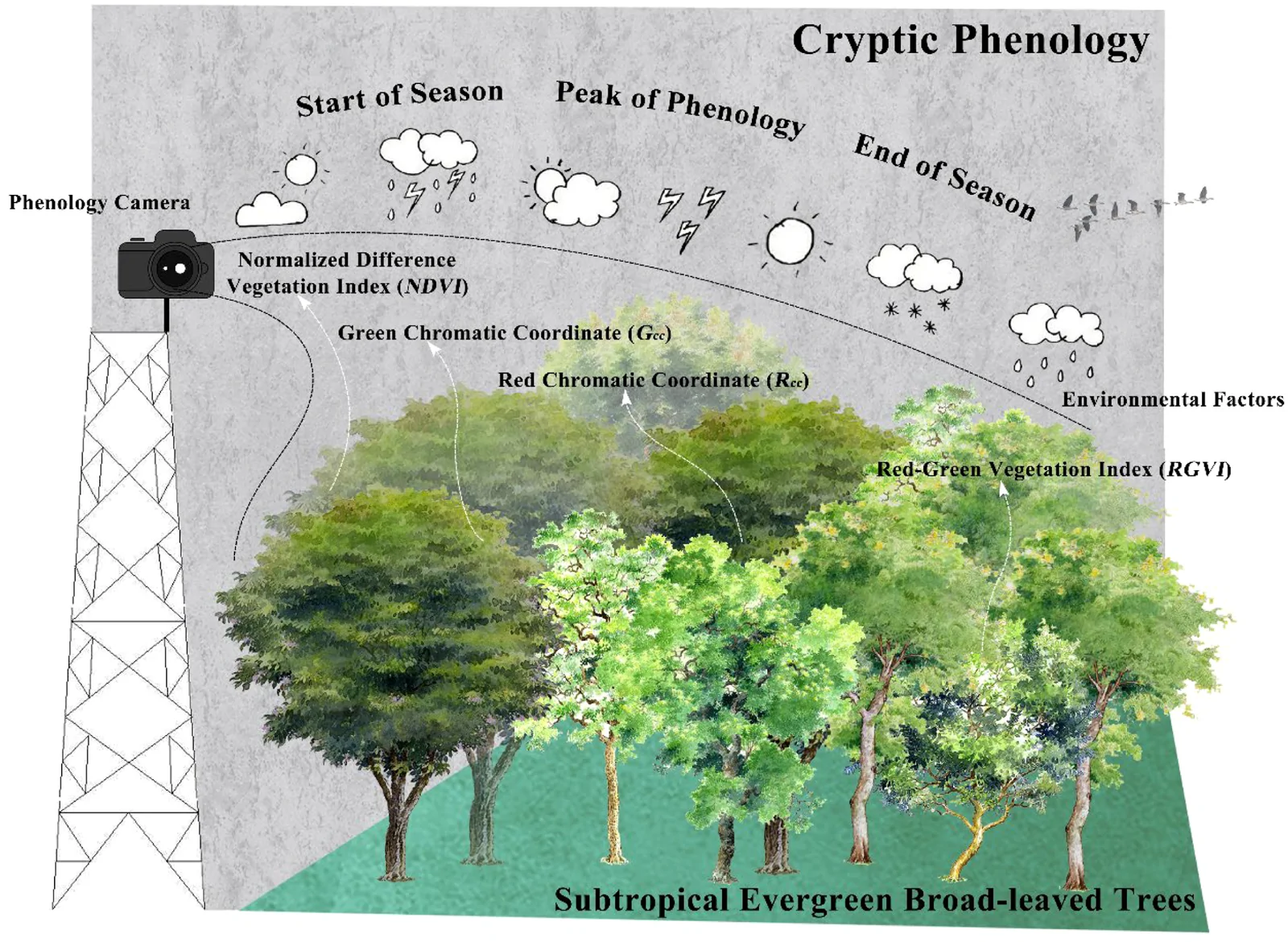

## 1 Introduction

Tree phenology, the timing of recurrent developmental events, provides a sensitive integrator of how plants respond to environmental variability across the annual cycle (Lieth, 1974). Key phenological phases [i.e., the Start of Season (*SOS*) and the End of Season (*EOS*)] are especially informative about climate–vegetation interactions and ecosystem functioning (Zhou, 2019). Phenological observations have traditionally emphasized visually observable events, including flowering, leaf senescence, and leaf fall (Piao et al., 2006), collectively termed as “apparent phenology”, which closely track seasonal environmental conditions (Leopold and Jones, 1947). In contrast, subtler, continuous changes in physiological activity, referred to as “cryptic phenology” (Albert et al., 2019), are captured by canopy vegetation indices like the Normalized Difference Vegetation Index (*NDVI*) and RGB-based chromatic indices, such as Green Chromatic Coordinate (*G_cc_*), Red Chromatic Coordinate (*R_cc_*) and Red-Green Vegetation Index (*RGVI*). Despite increasing use of these indicators, the mechanisms of canopy phenology in subtropical evergreen broad-leaved trees remain poorly revolved (Piao et al., 2019).

Because of their climate, evergreen broad-leaved trees in subtropical regions exhibit growth patterns distinct from those in northern China. Apart from the coldest part of winter, they remain in a growing state for most of the year (Du et al., 2019). Asynchronous leaf flushing and shedding create relatively uniform canopy dynamics and persistent greenness, making phase transitions less conspicuous to visual inspection. Consequently, robust characterization of their seasonality requires approaches that track changes in canopy physiological proxies, i.e. cryptic phenology.

Environmental factors such as air temperature, moisture and radiation regulate phenological timing in these systems (Chen et al., 2017). However, to date, systematic experiments in subtropical evergreen broad-leaved forests are limited. The relative importance of meteorological controls and the degree to which temperate observations can be extrapolated to subtropical regions remain unclear (Piao et al., 2019). Moreover, phenology often reflects antecedent (lagged) environmental effects. For example, accumulated warmth [growing degree days (*GDD*)] constrains the timing of spring budbreak and flowering (Montgomery et al., 2020; Walde et al., 2025); accumulated chilling modulates subsequent thermal requirements for tree budbreak (Walde et al., 2022); and precipitation history influences the onset and cessation of the growing season (Dixon et al., 2025). Accounting for such lags is therefore essential when attributing phenophase variability.

Advances in near-surface remote sensing now enable continuous, ecosystem-scale observation of canopy dynamics. Among them, phenology cameras (*PhenoCam*s) acquire high-frequency time-series imagery from which vegetation indices are derived (Yamashita et al., 2019), and have become a key observational technique in recent years (Meng et al., 2021). It is widely used to track seasonal changes in vegetation activity and structure (Inoue et al., 2014). *PhenoCam*s quantify dynamics in green vegetation cover, tree flowering phenology (Crimmins and Crimmins, 2008), snow cover (Julitta et al., 2014), and grassland phenology (Inoue et al., 2015), etc. They are widely applied in forest and agricultural phenology, with growing use in in wildlife monitoring (Jeganathan et al., 2024; Yang et al., 2023). Canopy vegetation indices derived from *PhenoCam*s provide robust indicators of seasonal color changes and canopy status, effectively capturing fine-scale dynamics in forest canopies (Li et al., 2021; Young et al., 2025). Moreover, *PhenoCam* researches are increasingly organized into coordinated networks, significantly advancing comparative and synthetic studies of canopy phenology across sites and biomes (Li et al., 2024; Wang et al., 2023; Zhu et al., 2022).

Given the characteristics of evergreen broad-leaved canopies, *NDVI*, *G_cc_*, *R_cc_* and *RGVI* are selected as *PhenoCam*-derived indicators of cryptic phenology. *NDVI*, the most widely used vegetation index, reflects temporal variation in canopy growth status (Wu et al., 2017) and offers high spatial coverage and long temporal monitoring for analyzing large-scale vegetation change (Li et al., 2023a). *G_cc_*, extracted from photographic imagery, quantifies greenness relative to overall brightness and is closely related to canopy photosynthesis. It is widely used to delineate phenological events (Richardson et al., 2007). Compared with Excess Green (*ExG*), *G_cc_* is generally more sensitive to green vegetation, enhances vegetation signals and effectively reduces interference from soil and shadow (Woebbecke et al., 1995), thereby better expressing phenological changes driven by climate and environmental variability (Sonnentag et al., 2012). *R_cc_*, a standardized red chromatic coordinate, captures changes in the red channel and is well suited to describing the timing of autumn color change in many deciduous forests (Crall et al., 2017). *RGVI* is a derived index from *G_cc_* and *R_cc_*, and has been demonstrated to effectively capture variations in the visible coloration of foliage, especially associated with winter leaf reddening and photoprotective processes (Chen et al., 2025).

Evergreen broad-leaved forests on Dagangshan Mountain, Jiangxi Province, China, are selected for this cryptic-phenology study, using long-term *in situ PhenoCam* observations. Materials and methods are presented in Section 2, followed by Results (Section 3), Discussion (Section 4) and Conclusions (Section 5).

## 2 Materials and methods

### 2.1 Study area

**Figure 1:**
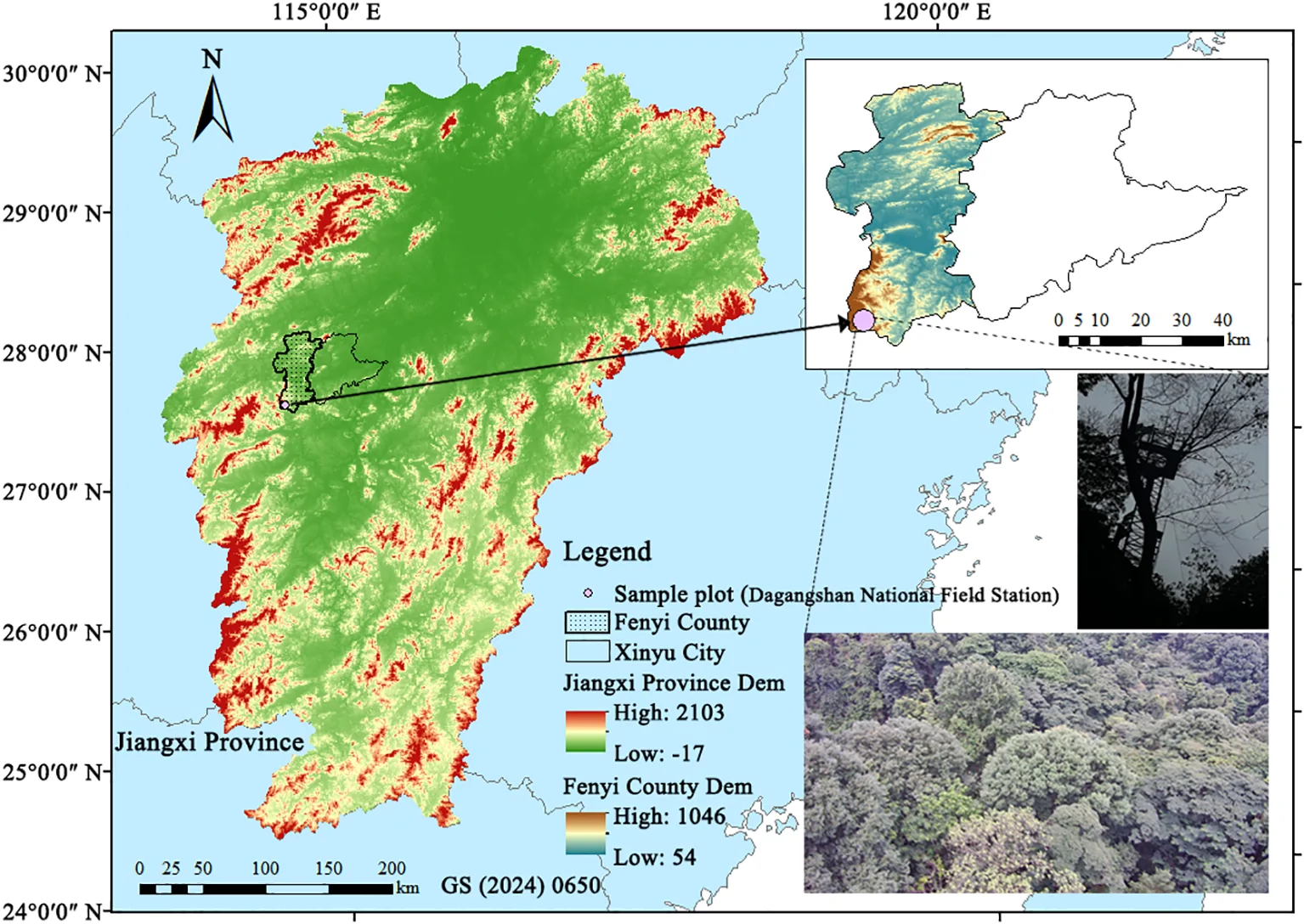
Study area and sample plot.

The study site is located at the Dagangshan National Key Field Observation and Research Station for Forest Ecosystem in Jiangxi Province, China (**Figure 1**, 27°30′ - 27°50′N, 114°30′ - 114°45′E), referred to as the Dagangshan National Field Station. The station occupies a branch of the Wugong Mountain Range at the northern end of the Luoxiao Mountains. The topography slopes from high ground in the west to lower elevation in the east, with a relief of ~1000 m and a maximum elevation of 1091.8 m. The climate is a mid-subtropical humid monsoon, with a mean annual temperature of 16.8 °C (July mean 28.8 °C; January mean 5.2 °C). Average annual sunshine duration is 1657.0 h, total solar radiation averages 486.6 kJ/cm², and annual precipitation average 1590.9 mm. The average annual evaporation is 1503.8 mm. Soils are predominantly red soil (Cui et al., 2006; Qiao et al., 2020; Xu et al., 2023).

The evergreen broad-leaved forest at Dagangshan National Field Station boasts a rich flora, with dominant tree species including *Castanopsis fargesii* and *Triadica cochinchinensis Loureiro* (Bai et al., 2021; Wang et al., 2020). Owing to its location and protection status, the forest is minimally affected by direct human disturbance, providing near-natural conditions well suited to ecological observation and phenological research.

### 2.2 Plot setting and tree species selection

**Figure 2:**
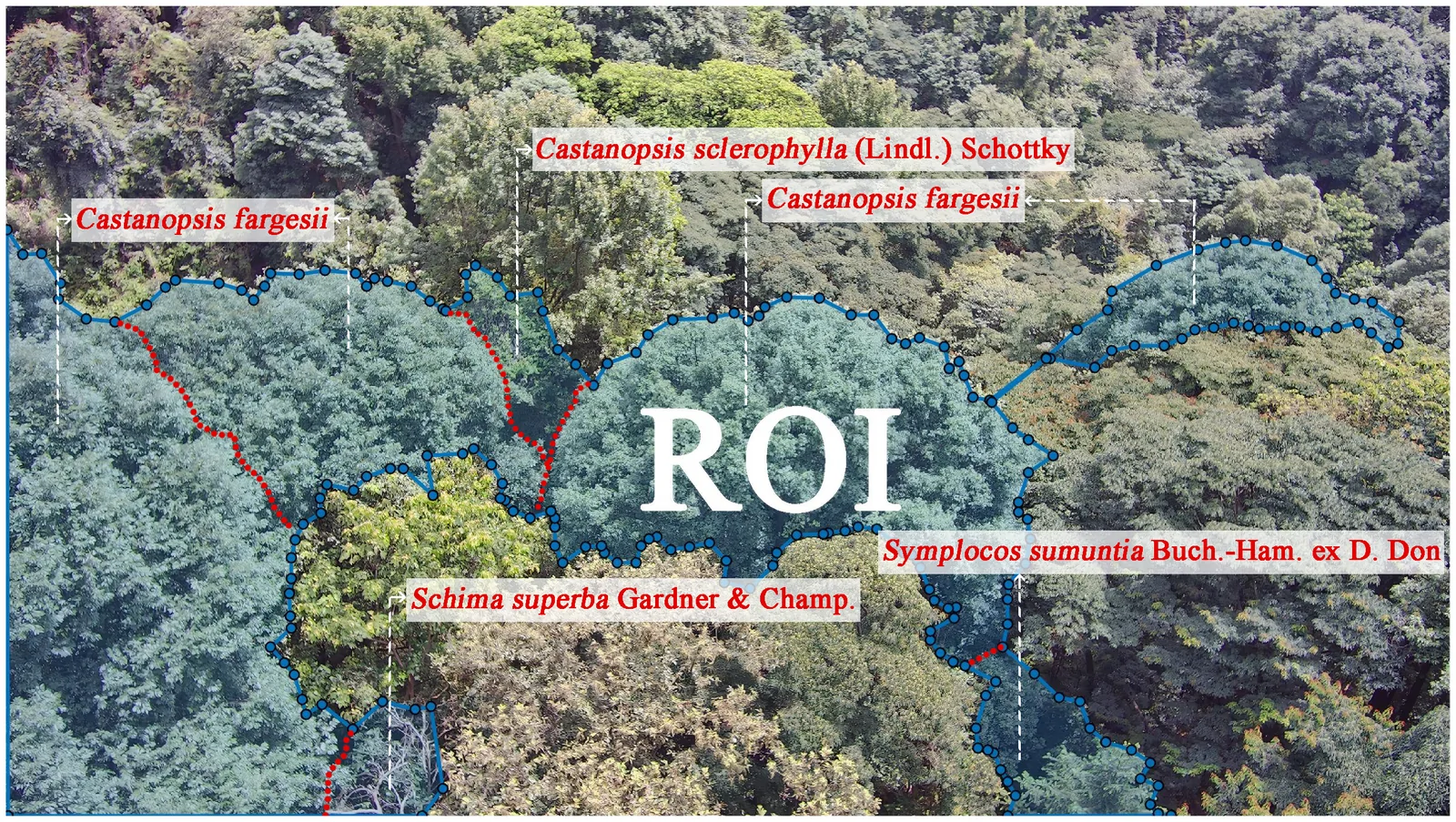
Spatial distribution of tree species and ROI delineation based on PhenoCam images.

This study was conducted in 2024. A 50 m × 50 m sample plot was established adjacent to the Evergreen Broadleaved Forest Comprehensive Observation Tower at the Dagangshan National Field Station. In accordance with the National Standard of the People’s Republic of China “Methodology for long-term observation of forest ecosystem (GB/T 33027-2016)“, white PVC pipes were installed at the four plot corners as fixed markers, and a full tree species survey was conducted. For *PhenoCam* observations, evergreen broad-leaved communities were selected in this study, taking into account camera view geometry (observation angle, field of view and range) and stand structure (the number and height of the dominant mature trees, typically ~20-30 m depending on growth habit). The species included in each community are listed in **Table 1** and the spatial locations of these species are shown in **Figure 2**.

**Table 1:** Basic information of evergreen broad-leaved trees in the sample plot.

Tree species	Number of trees
*Castanopsis fargesii*	4
*Castanopsis sclerophylla* (Lindl.) Schottky	1
*Symplocos sumuntia* Buch.-Ham. ex D. Don	1
*Schima superba* Gardner & Champ.	1

### 2.3 Data acquisition

The primary data for this study are *NDVI*, *G_cc_*, *R_cc_* and *RGVI* observed by the plant growth rhythm phenology camera (*PhenoCam*) mounted on the top of the evergreen broad-leaved forest comprehensive observation tower in 2024. There are 14 meteorological variables measured at the ground standard meteorological observation field the Dagangshan National Field Station during 2023-2024, including: air temperature (*T_air_*), relative humidity (*RH*), solar radiation (*SR*), wind speed (*WS*), precipitation (*P*), soil temperature (*T_soil_*), soil moisture content (*SMC*), net radiation (*NR*), ultraviolet radiation (*UV*), atmosphere pressure (*Atm*), photosynthetically active radiation (*PAR*), evapotranspiration (*Evap*), water level (*WL*) and vapor pressure deficit (*VPD*).

**Figure 3:**
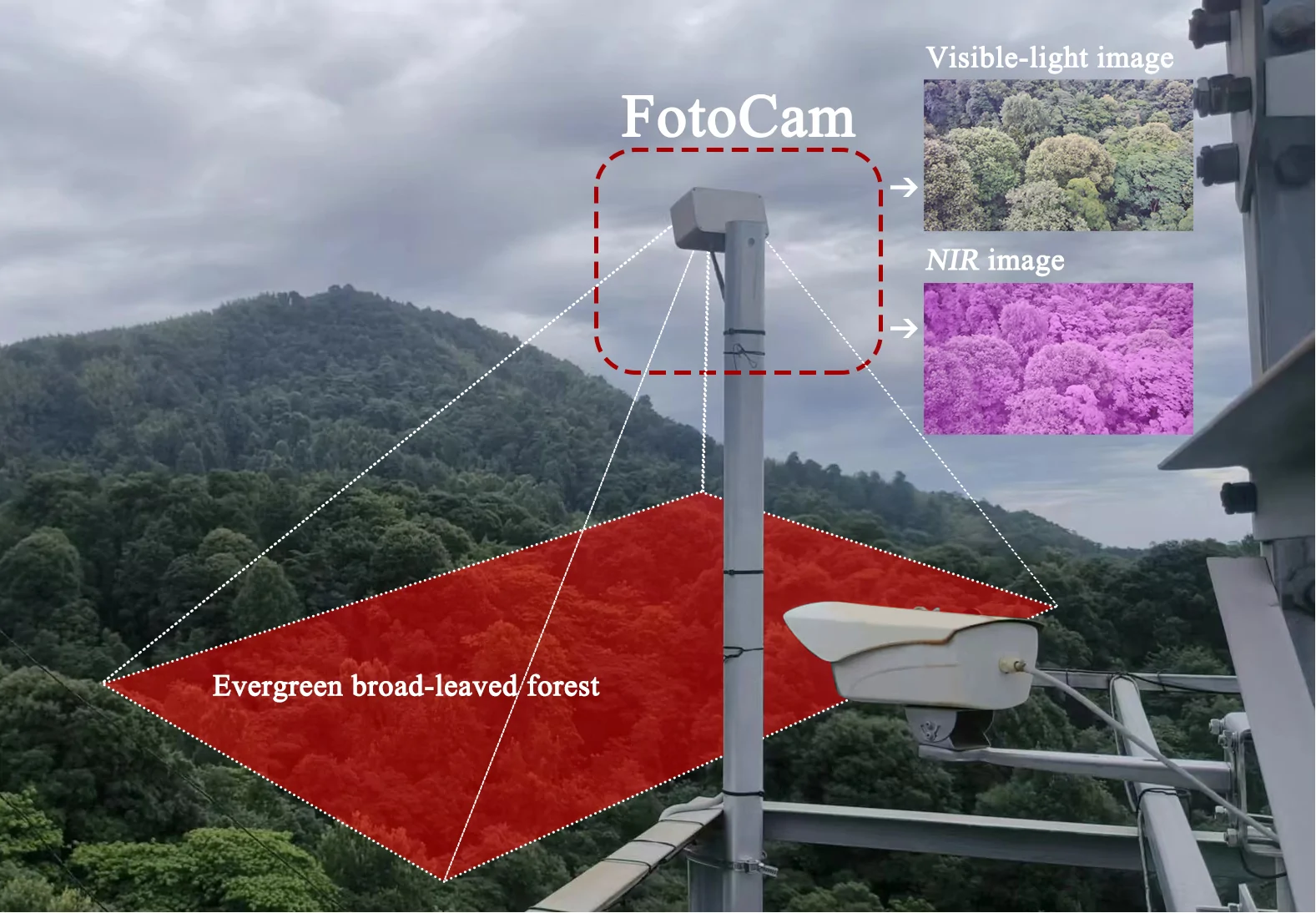
Schematic diagram of FotoCam setup at the Dagangshan National Field Station’s evergreen broad-leaved forest integrated observation tower.

The *PhenoCam* (FotoCam PA200, Beijing Tianhang Huachuang Technology Co., Ltd., Beijing, China) was installed at a height of 30 m with a depression angle of approximately 30° to capture the target canopy (**Figure 3**). Four sets of images were acquired daily at 8:10 a.m., 10:10 a.m., 2:10 p.m. and 4:10 p.m. local time at 4K (3840 × 2160) resolution, with each acquisition consisting of two image types: visible-light and near-infrared (*NIR*) images. Meteorological variables were monitored in real time using a standard ground-based meteorological observation station. All sensors were connected to a data logger, with measurements recorded at 10-minute intervals.

### 2.4 Data processing

#### 2.4.1 Extraction of *NDVI* and RGB-based chromatic indices

Automated *PhenoCam* image analysis enables extracting quantitative color information from the visible bands (red (*R*), green (*G*), and blue (*B*)) and *NIR* (Petach et al., 2014). These data were subsequently transformed into vegetation indices indicating canopy status, including *NDVI*, *G_cc_*, *R_cc_* and *RGVI* (Gillespie et al., 1987).

Image processing began with delineation of regions of interest (*ROI*) (Knox et al., 2017), the within-image areas from which *RGB* and *NIR* statistics were extracted (Richardson et al., 2018). Using MATLAB R2023b, *ROI*s were digitized on the canopy phenology photographs using the mouse (**Figure 2**). To reduce sensitive short-term variability of time series caused by illumination and viewing geometry, the 90th percentile (*P90*) method proposed by Sonnentag et al. (2012) was applied. Specifically, four *PhenoCam* images were acquired each day (08:10 a.m., 10:10 a.m., 14:10 p.m., and 16:10 p.m.), from which vegetation indices were individually extracted. These daily observations were then organized using a three-day moving window, within which all values were pooled and the *P90* value was selected as the representative value for the central day. This procedure was applied sequentially to generate a continuous daily time series. This procedure was applied sequentially to generate a continuous daily time series in 2024. For each *ROI*, NDVI, *G_cc_ R_cc_* and *RGVI* were calculated based on the red, green, blue and *NIR* bands for each pixel within the *ROI* according to Equations 1–3 as follows:

(1) $$NDVI=\frac{NIR_{DN}-R_{DN}}{NIR_{DN}+R_{DN}}$$

Where *R_DN_* and *NIR_DN_* are the mean pixel values of the red band extracted from the visible light image *ROI* and the *NIR* band extracted from the *NIR* image *ROI*, respectively. *DN* is the digital number.

(2) $$G_{cc}=\frac{G_{DN}}{R_{DN}+G_{DN}+B_{DN}}$$

(3) $$R_{cc}=\frac{R_{DN}}{R_{DN}+G_{DN}+B_{DN}}$$

Where *G_DN_* and *B_DN_* are the mean values (intensity measures) of the green and blue digital values within the *ROI*, respectively. Similarly, the Blue Chromatic Coordinate (*B_cc_*) is defined as the standardized blue digital value and can also be obtained similar to Equation 2, 3. *RGVI* calculated to quantify vegetation greenness based on the difference between *G_cc_* and *R_cc_*, as shown in Equation 4 (Chen et al., 2025). The time sequence of the above data results was expressed as “Day-of-Year” (*DOY*) (Zhang et al., 2003).

(4) $$RGVI=\frac{R_{cc}-G_{cc}}{R_{cc}+G_{cc}}$$

Above all, *NDVI*, *G_cc_*, *R_cc_*, and *RGVI* were smoothed using the Savitzky-Golay (*SG*) filter with a nine-point sliding window.

#### 2.4.2 Statistical analysis of environmental data

This study used principal component analysis (*PCA*) to perform dimensionality reduction analysis on environmental factor data. Principal component analysis is a multivariate statistical technique that uses an orthogonal transformation to transform a set of correlated variables into a set of orthogonal, uncorrelated axes, reducing the spatial dimensionality of the data. It is the most commonly used technique for identifying linear combinations of high-dimensional variables (Yao et al., 2012). Fourteen meteorological factors monitored at the Dagangshan National Field Station in 2024 were included. *PCA* was performed in Origin 2021 software and the resulting component scores were treated as principal component (*PC*) environmental factors.

Since vegetation indices reflect canopy condition and growth, and the growth is influenced by multiple environmental factors, the strength of these relationships was quantified. Pearson correlation analysis was used to: (i) assess intercorrelations among the meteorological variables; and (ii) evaluate associations between *NDVI*, *G_cc_*, *R_cc_* and *RGVI* and both the original variables and the *PC* scores. In combination with *PCA* loadings, these correlations were used to identify the primary environmental factors and to gauge their relative influence on *NDVI*, *G_cc_*, *R_cc_* and *RGVI*.

#### 2.4.3 Phenology simulation based on double logistic model

To accurately extract key phenological parameters of the vegetation in the study area, annual *NDVI*, *G_cc_*, *R_cc_* and *RGVI* time series data were assembled and then fitted with a double Logistic function to identify key phenological events in 2024. The double logistic function modeled the full annual cycle by superimposing two logistic curves, thereby the spring greening-up, summer growth peak phase and autumn senescence phase as a continuous trajectory. This method is well suited to capturing the dynamics of the vegetation greening season and identifying the start and end points of the greening season (Zhang et al., 2004a). Parameter estimation was performed via nonlinear least squares fitting in MATLAB, and the following typical phenological indicators were derived from the fitted curves: *SOS* (Start of Season), the onset of a significant increase in spring greening; *POP* (Peak of Phenology), the period of the strongest vegetation activity during the growing season; *EOS* (End of Season), the starting point of a significant decline in autumn decline; and *LOS* (Length of Season), the time span between *SOS* and *EOS*.

Specifically, *SOS* and *EOS* were determined based on the rate of change of the fitted curves. *SOS* was defined as the date corresponding to the maximum positive first derivative during the spring green-up phase, while *EOS* was defined as the date corresponding to the maximum negative first derivative during the autumn senescence phase, i.e., the inflection points of the double logistic function. *POP* was defined as the date of the maximum fitted value, and *LOS* was calculated as the difference between *EOS* and *SOS*. Curvature-based inflection points shifted irregularly, at times increasing and then decreasing, which, without correction, would invert *SOS* and *EOS*. Therefore, the derivative-based extraction within the double logistic framework was adopted in this study to ensure robust and consistent identification of phenological transition dates.

The functional form used for modeling is given in Equation 5:

(5) $$y(t)=y_{0}+\frac{a_{1}}{1+e^{-k_{1}(t-t_{1})}}+\frac{a_{2}}{1+e^{-k_{2}(t-t_{2})}}$$

where *y*(*t*) represents the *NDVI*, *G_cc_*, *R_cc_* or *RGVI* value corresponding to day *t* in *DOY*; *y*_0_ is the background value in the non-growing season; *a*_1_, *k*_1_ and *t*_1_ represent the amplitude, rate and inflection point of the spring greening process, respectively; *a*_2_, *k*_2_, and *t*_2_ correspond to the amplitude, rate, and inflection point of the autumn decline process, respectively.

#### 2.4.4 Environmental lag effect calculation

Because plant phenology is affected by environmental lag effects from the previous quarter (Fu et al., 2024; Ma et al., 2024), this study selects several environmental driving factors from the preceding year (2023) and the study year (2024) that have a significant impact on *SOS*, *POP* and *EOS* (**Table 2**). The detailed Equations and references (Refs.) are provided below, shown as Equations 6-14.

**Table 2:** Driving factors and calculation principles of different key phenological events.

Key phenological events	Driving factors	Equations	Calculation instructions	*Refs.*
*SOS*	*GDD*	(6)	Cumulative value; Based on the characteristics of the subtropical climate, *T_base_* in this study is set at 5°C; the window length is from January 1st of the year to *SOS*.	(Kim et al., 2014; Shi et al., 2025)
	Dynamic *CP*	(7)	A dynamic model (a model that accumulates chilling and forcing to predict bud break or the start of the growing season) is used; the window length is from the beginning of winter in the previous year to the *SOS* of the current year.	(Campoy et al., 2011)
	Spring *PAR*	(8)	Cumulative value; the window length is from January 1st of the current year to *SOS*.	(Qin et al., 2023)
*POP*	*Temp* stability	(9)	The sample standard deviation formula is used; the window length is 30 days before *POP* (Deng et al., 2025)	(Bai and Li, 2022)
	Precipitation	(10)	Cumulative value; the window length is 30 days before *POP*	(Chen et al., 2020)
	Mean *SMC*	(11)	Mean value; the window length is 30 days before *POP*	(Jing et al., 2025)
	Mean *PAR*	(13)	Mean value; the window length is 30 days before *POP*	(Liu et al., 2025)
*EOS*	*T_mean_*	(14)	Mean value; the window length is the first 30 days of *EOS*	(Qi et al., 2023)
	*CDD*	(12)	Cumulative value; Based on the subtropical climate characteristics, *T_base_* in this study is set at 20°C as the comfortable temperature; the window length is 30 days before *EOS*	(Dicko et al., 2024)
	Mean *PAR*	(13)	Mean value; the window length is the first 30 days of *EOS*	(Yuan et al., 2022)
	Precipitation	(10)	Cumulative value; the window length is the first 30 days of *EOS*	(Lai et al., 2023)
	Mean *SMC*	(11)	Mean value; the window length is the first 30 days of *EOS*	(Cui et al., 2022)

(1) Growing Degree Days (*GDD*):

(6) $$GDD=\sum max(T_{avg}-T_{base})$$

Where *T_avg_* is the daily average temperature ( $T_{avg}=\frac{T_{max}+T_{min}}{2}$), which is the mean of the highest (*T_max_*) and lowest (*T_min_*) temperatures of the day; *T_base_* is the base temperature (McMaster and Wilhelm, 1997).

(2) Dynamic Chill Portions (Dynamic *CP*):

(7) $$CP_{day}=0.04\times exp[-0.5\times {\left(\frac{T-T_{opt}}{\sigma }\right)}^{2}]\times \frac{1}{1+\text{exp}(T-18)}$$

Where *T* is the daily average temperature, *T_opt_* is the optimum temperature, and *σ* is the standard deviation of the temperature response (Luedeling et al., 2009).

(3) Spring PAR:

(8) $$PAR_{spring}=\sum PAR(t)\times \Delta t$$

*PAR*(*t*) is the photosynthetically active radiation intensity at time *t*, and Δ*t* is the time interval usually in days (Gamon and Surfus, 1999).

(5) Temperature (*Temp*) stability:

(9) $$\sigma_{T_{a}}=\sqrt{\frac{1}{n-1}\sum_{i=1}^{n}{\left(T_{a,i}-{\bar{T}}_{a}\right)}^{2}}$$

Where *T_a,i_* is the temperature on the *i*-th day, ${\bar{T}}_{a}$ is the average temperature, and *n* is the number of data points (Whiting, 1978).

(6) Sum of precipitation:

(10) $$Prcp_{sum}=\sum Prcp(t)$$

Where *Prcp*(*t*) is the precipitation at time *t* (Hobbins et al., 2001).

(7) Mean *SMC*

(11) $$SMC_{mean}=\frac{1}{n}\sum_{i=1}^{n}SMC_{i}$$

Where *SMC_i_* is the soil moisture content on the *i*-th day, and *n* is the number of days in the period (Zhang et al., 2004b).

(8) Cooling Degree Days (*CDD*):

(12) $$CDD=\sum max(T_{avg}-T_{base})$$

Where *T_avg_* is the daily average temperature and *T_base_* is the comfortable temperature (Krese et al., 2012).

(9) Mean *PAR*

(13) $$PAR_{mean}=\frac{1}{n}\sum_{i=1}^{n}PAR_{i}$$

Where *PAR_i_* is the *PAR* of the *i*-th day, and *n* is the number of days in the period (Allen et al., 1998).

(10) Mean temperature (*T_mean_*)

(14) $$T_{mean}=\frac{1}{n}\sum_{i=1}^{n}T_{i}$$

Where *T_i_* is the temperature on the *i*-th day, and *n* is the number of days in the period (Monteith and Unsworth, 2013).

## 3 Results

### 3.1 Trends in *NDVI*, *G_cc_*, *R_cc_* and *RGVI*

**Figure 4:**
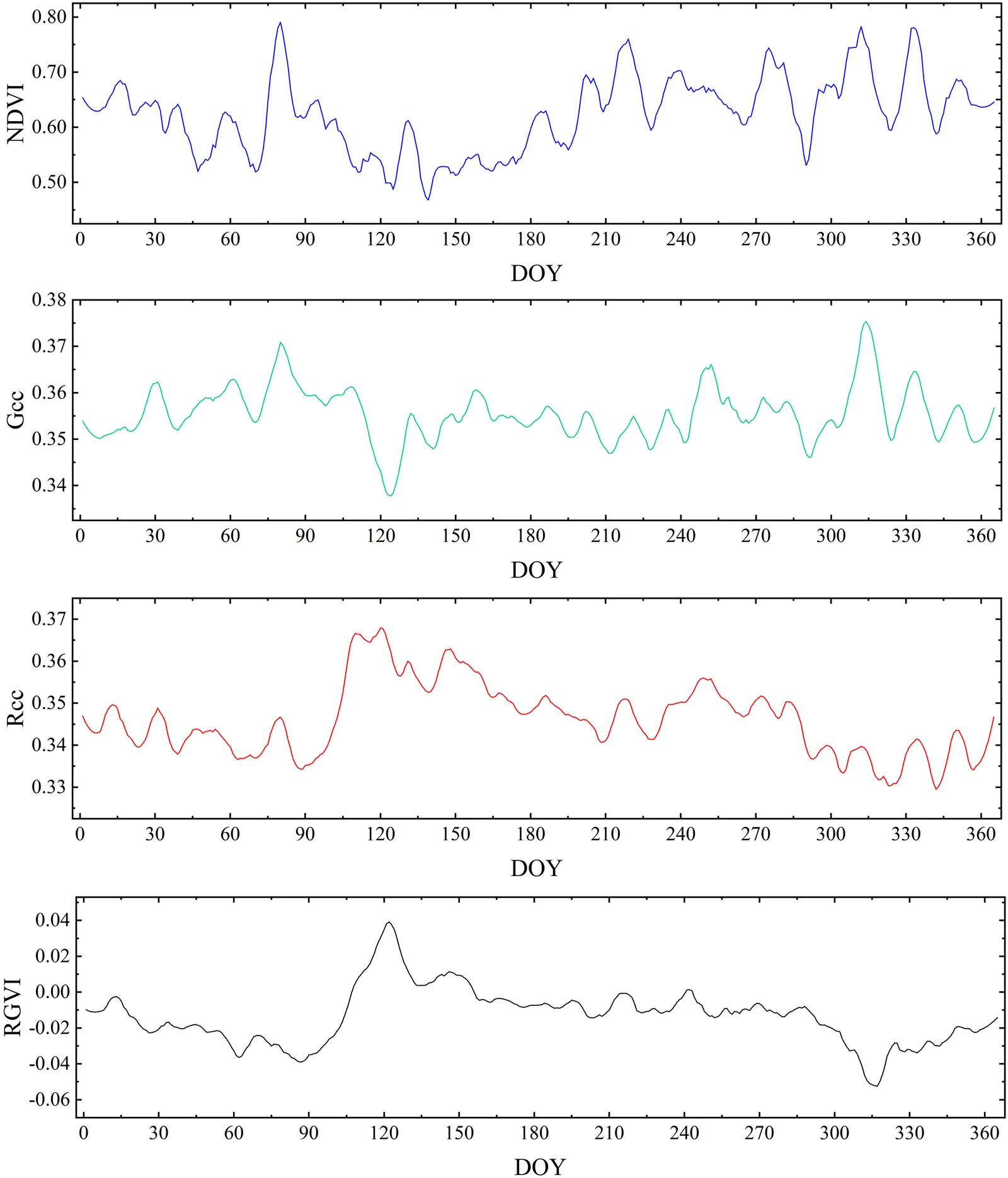
*NDVI*, *G_cc_*, *R_cc_* and *RGVI* of evergreen broad−leaved trees derived from *PhenoCam* observations throughout 2024.

Based on *PhenoCam* image extraction, the trends of *NDVI*, *G_cc_*, *R_cc_*, and *RGVI* of 2024 are shown in **Figure 4**. With the exception of a few extreme values, *NDVI* showed little pronounced intra-annual variation, and Values were generally greatest through mid-summer (July – August), indicating optimal tree growth during this period. Consistent with a subtropical evergreen broad-leaved canopy, *NDVI* shows weak seasonality yet remains high, generally ~ 0.60–0.80, with observed extremes of 0.468 (*DOY* 139) and 0.790 (*DOY* 80), indicative of persistent foliage. *G_cc_* likewise exhibited weak seasonality, fluctuating between 0.3333 (*DOY* 124) and 0.3754 (*DOY* 314). By contrast, *R_cc_* displayed a pronounced seasonal peak, rising from winter into the warm season and declining thereafter - with a minimum of 0.3295 (*DOY* 342) and a maximum of 0.3679 (*DOY* 120). *R_cc_* increased at a mean rate of 0.001 per day from 30 March to 29 April (*DOY* 90–120), indicating peak canopy activity during early spring. It then showed limited intra-seasonal variability from 29 May to 26 September (*DOY* 150-270), remaining at a consistently high level (0.341-0.361). From 27 September to 5 November (*DOY* 271-310), *R_cc_* declined at a mean rate of 0.0003 per day, consistent with a gradual slowdown in growth. *RGVI* exhibited a markedly stronger seasonal signal compared with *NDVI* and *G_cc_*, and a more pronounced dynamic range than *R_cc_*. Values were predominantly negative throughout the year, with a minimum of -0.0525 (*DOY* 317), and became positive only during the peak growing period. *RGVI* increased rapidly in early spring (*DOY* 95-110), crossing zero near *DOY* 107 and reaching a maximum of 0.0391 (*DOY* 122). During this period, *RGVI* increased at a mean rate of approximately 0.003 per day from 4 April to 1 May (*DOY* 95-122), indicating rapid canopy development. Thereafter, values declined, returning to negative in *DOY* 155 and remaining relatively stable during summer and mid-autumn until around *DOY* 289. From *DOY* 289 to 315, *RGVI* declined at a mean rate of approximately 0.0016 per day from 15 October to 10 November, indicating a rapid decrease in canopy activity during late autumn.

Comparing the four series, *NDVI* and *G_cc_* co-varied closely and exhibited only weak and relatively stable seasonal variation, whereas *R_cc_* and especially *RGVI* showed more pronounced seasonal dynamics, although a general correspondence among all indices was still evident.

### 3.2 Effects of environmental factors on canopy phenology of evergreen broad-leaved trees

Environmental factors exert varying degrees of influence on tree canopy phenology, and this influence is rarely singular. Because environmental factors are interrelated, it is necessary to identify the primary factors influencing evergreen broad-leaved tree canopy phenology.

**Figure 5:**
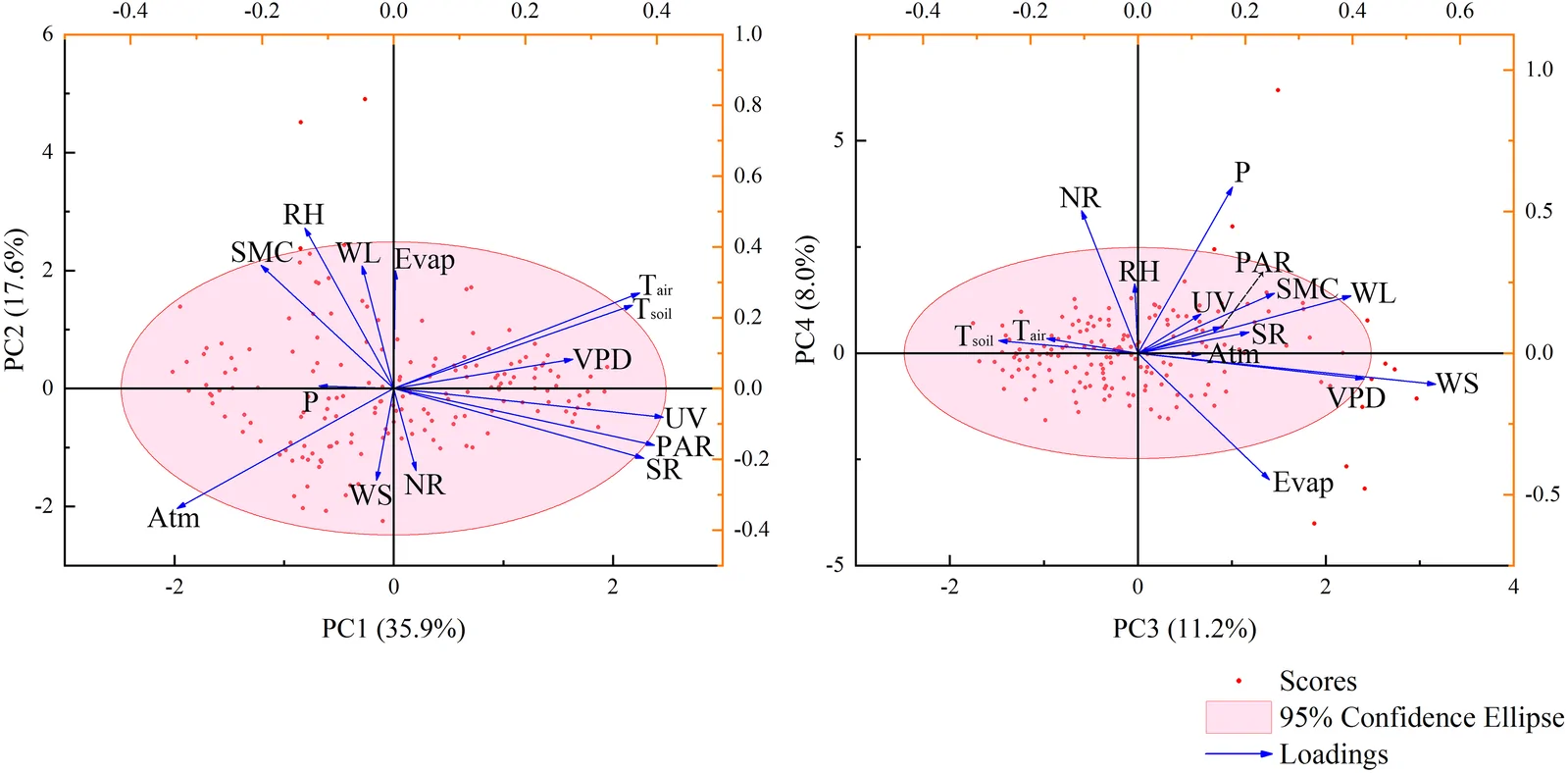
*PCA* of environmental factors.

**Figure 6:**
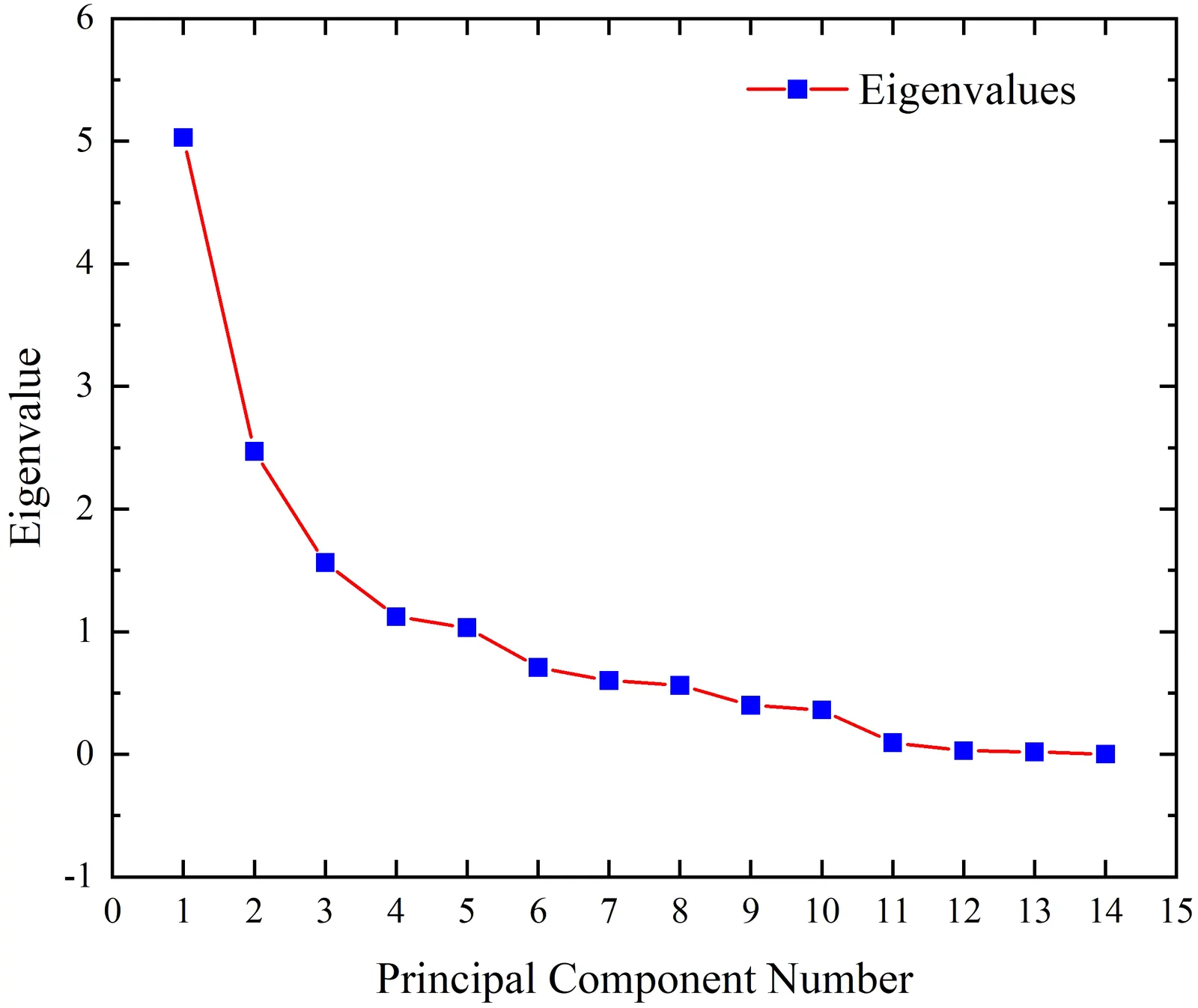
Scree plot of *PCA* of environmental factors.

As shown in **Figure 5**, *PCA* was performed for dimensionality reduction analysis on 14 environmental factors collected from meteorological fields within the study area in 2024 (366 data samples per variable). Based on **Figure 6** and a cumulative variance contributions exceeding 70%, four *PC*s were selected which explained 35.9%, 17.6%, 11.2% and 8.0% of the total variance, respectively, for a cumulative contribution of 72.7% (Jolliffe, 2005), effectively capturing the primary information of these environmental factors. The loadings of each environmental factor on the four *PC*s are shown in **Table 3**, with higher loadings indicating higher contributions.

**Table 3:** Environmental factors’ loadings in four PCs.

Environmental factors	*PC*1	*PC*2	*PC*3	*PC*4
*T_air_*	0.374	0.270	−0.170	0.052
*RH*	−0.135	0.454	−0.006	0.244
*SR*	0.380	−0.197	0.207	0.073
*WS*	−0.026	−0.260	0.555	−0.110
*P*	−0.114	0.007	0.176	0.587
*T_soil_*	0.363	0.236	−0.259	0.044
*SMC*	−0.202	0.348	0.254	0.212
*NR*	0.034	−0.232	−0.105	0.503
*UV*	0.410	−0.081	0.118	0.138
*Atm*	−0.329	−0.339	0.117	−0.004
*PAR*	0.396	−0.160	0.156	0.092
*WL*	−0.048	0.347	0.397	0.202
*Evap*	0.003	0.333	0.245	−0.447
*VPD*	0.272	0.083	0.421	−0.090

**Figure 7:**
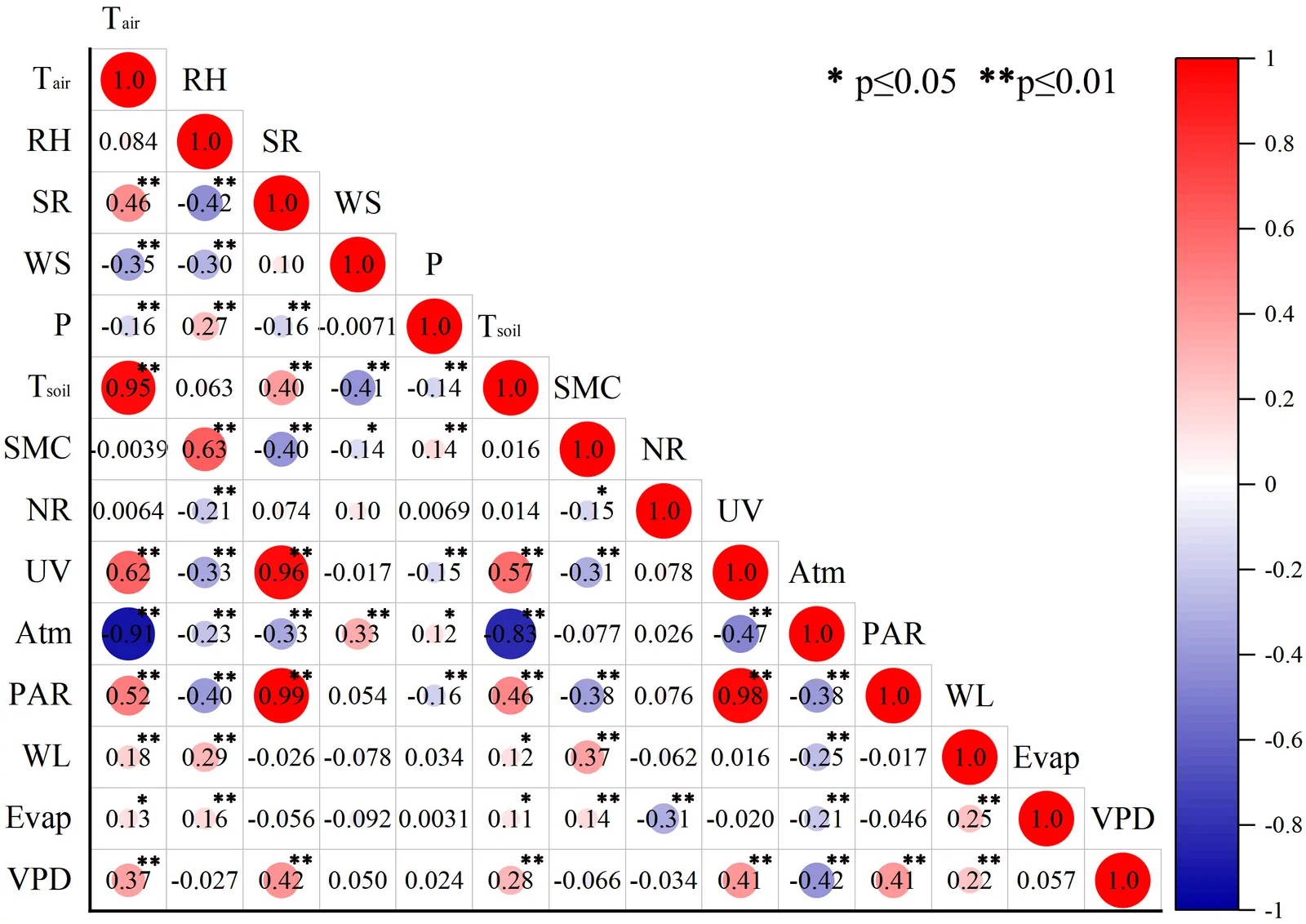
Correlation between 14 environmental factors.

Within each principal component, only variables with a loading greater than 0.3 (in absolute value) were considered to have explanatory significance. On this basis, and combined with Pearson correlation analysis of the 14 environmental factors (**Figure 7**), which was used to evaluate inter-variable correlations and identify potential redundancy prior to PCA, redundant highly correlated variables within each principal component were eliminated, and variables with greater ecological significance within the same category were selected. Four environmental factor patterns were identified. Representative environmental factors within *PC*1 are *UV*, *PAR*, *SR*, *T_air_* and *T_soil_*, so *PC*1 can be interpreted as a “radiation-temperature” factor. Similarly, representative factors within *PC*2 are *RH*, *SMC*, *WL* and *Atm*, so *PC*2 is a “moisture-pressure” factor. Representative factors in *PC*3 and *PC*4 are *WS* and *P*, respectively, so *PC*3 and *PC*4 correspond to “wind speed” and “rainfall,” respectively.

**Figure 8:**
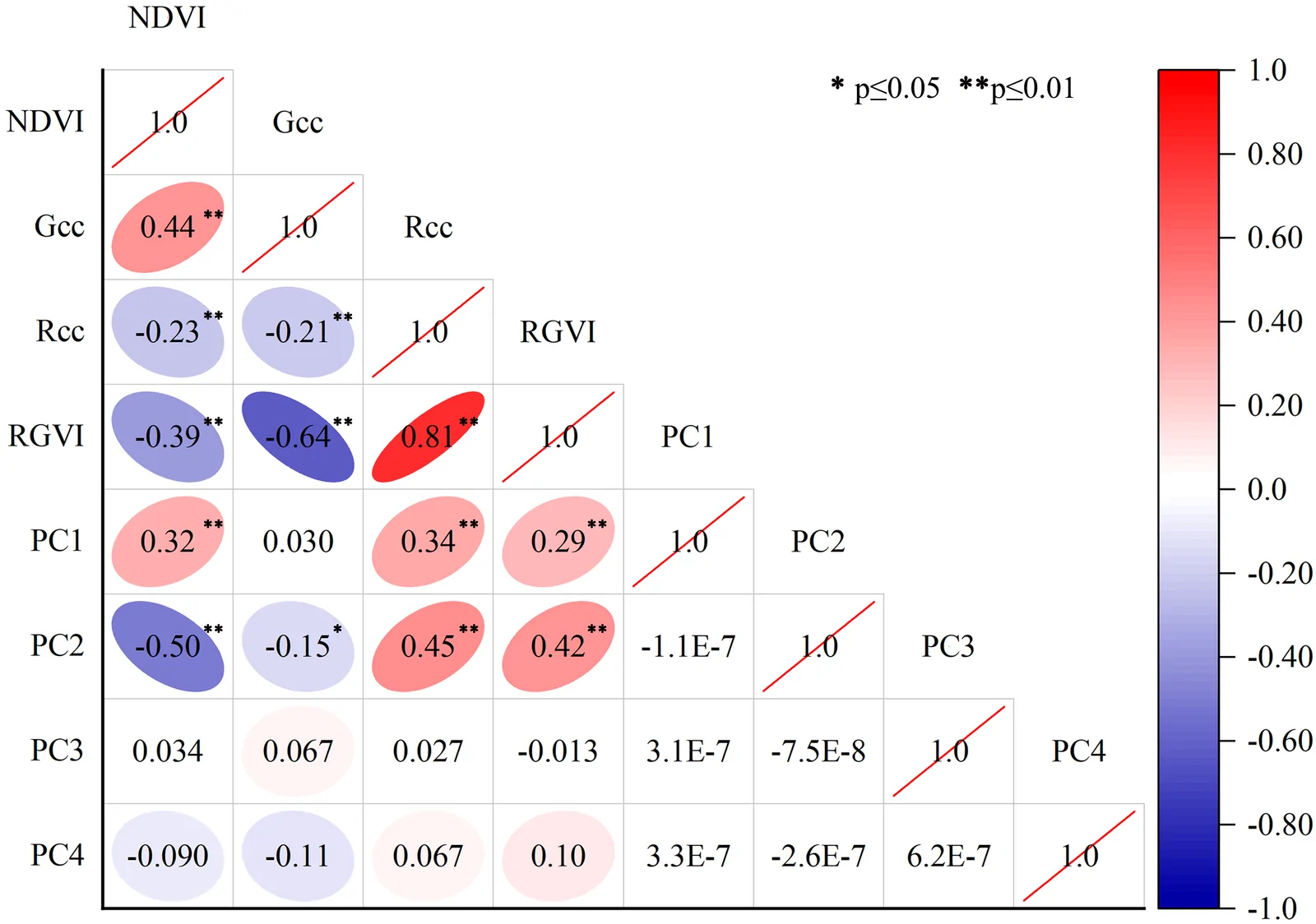
Correlation between phenological indices and several major environmental pattern factors.

As shown in **Figure 8**, Pearson correlation analysis was performed between the obtained principal components and *NDVI*, *G_cc_*, *R_cc_* and *RGVI*. Here, r represents the Pearson correlation coefficient, with |r| values closer to 1 indicating stronger correlations; *p* ≤ 0.05 and *p* ≤ 0.01 denote statistical significance at the 5% and 1% levels, respectively. The four canopy phenology indices were strongly correlated, with *R_cc_* and *RGVI* negatively correlated with both *G_cc_* and *NDVI* (*p* < 0.01). *NDVI*, *G_cc_*, *R_cc_* and *RGVI* all correlated with *PC*1 and *PC*2, whereas associations with *PC*3 and *PC*4 were weaker. *NDVI* showed a highly significant positive correlation with *PC*1 (*r* = 0.32, *p* < 0.01) and a highly significant negative correlation with *PC*2 (*r* = -0.50, *p* < 0.01). *G_cc_* showed no correlation with *PC*1 (*r* = 0.03, *p* => 0.05) and a weak but significant correlation with *PC*2 (*r* = 0.15, *p* < 0.05). *R_cc_* showed highly significant positive correlations with both *PC*1 (*r* = 0.34, *p* < 0.01) and *PC*2 (*r* = 0.45, *p* < 0.01). *RGVI* showed similarly to *R_cc_*, with greatly significant positive correlations with *PC*1 (*r* = 0.29, *p* < 0.01) and *PC*2 (*r* = 0.42, *p* < 0.01).

In short, canopy phenology in this study is mainly associated with radiation, temperature, moisture and air pressure. However, due to the discontinuity of subtropical rainfall and the influence of some extreme rainy-season events, rainfall and wind speed do not appear to play a key role.

### 3.3 Determination of canopy phenology of subtropical evergreen broad-leaved trees

After fitting *NDVI*, *G_cc_*, *R_cc_* and *RGVI* time series data in 2024 with the double logistic model, the *NDVI*- and *G_cc_*-based curves did not yield stable transition points, indicating challenges in directly using curvature-based metrics for phenological extraction. This behavior is consistent with the weak seasonality of subtropical evergreen canopies and with the weaker or negative associations noted in the correlation analysis.

By contrast, although the fitting of *R_cc_* yields apparent rising and falling inflection points, the robustness of the derived phenological estimates is limited by the small dynamic range of the *R_cc_* time series. *R_cc_* shows only minor short-term fluctuations (approximately 0.01) relative to its constrained annual amplitude (about 0.02), which increases the sensitivity of the fitting procedure and consequently reduces the reliability of the estimated phenological timings.

**Figure 9:**
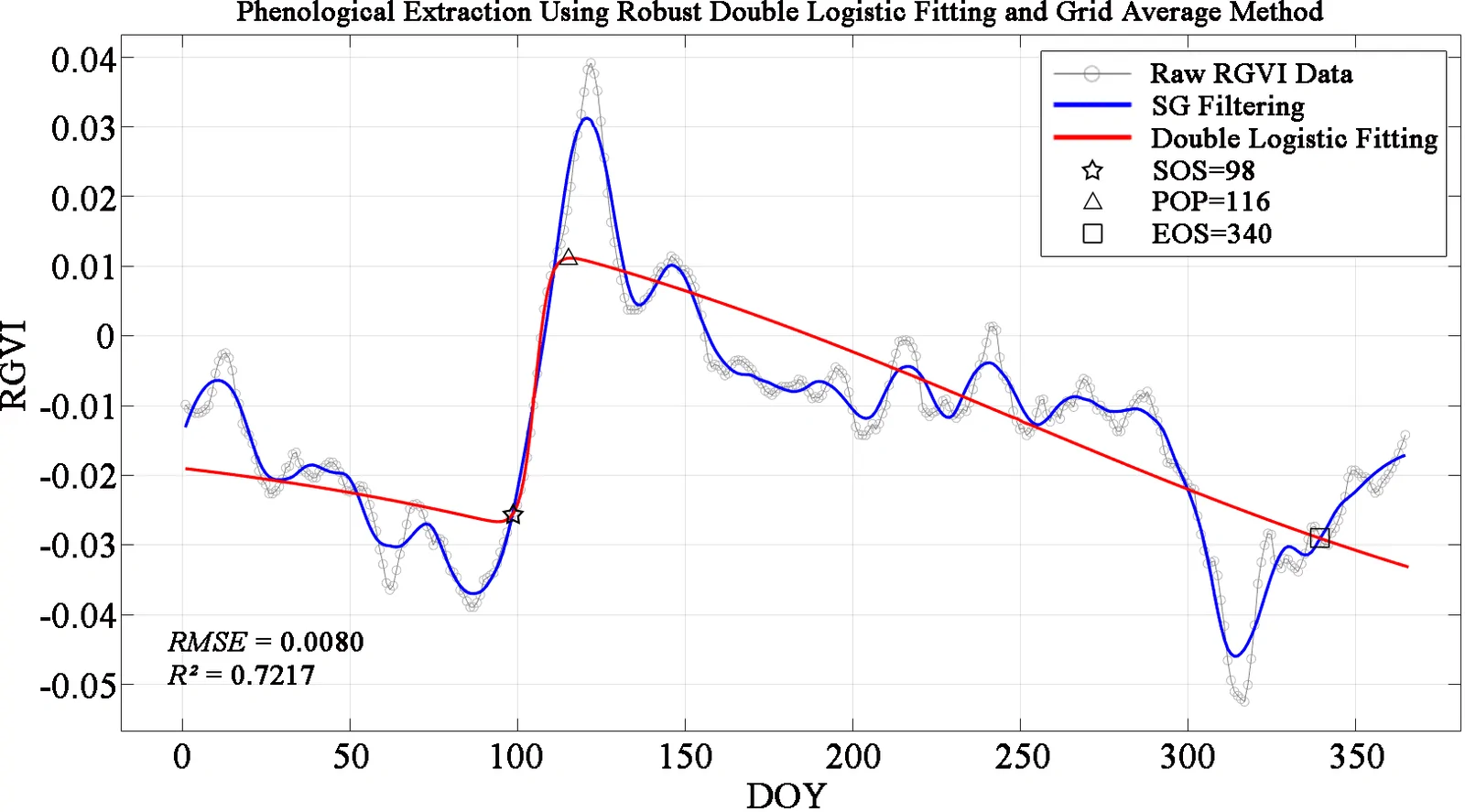
*RGVI* simulation results based on double logistic.

Based on its demonstrated robustness in phenological monitoring, *RGVI* was used as the primary index for double logistic fitting in this study, the fitting results are shown in **Figure 9**. The extracted dates were: *SOS* = *DOY* 98 (7 April), *POP* = *DOY* 116 (25 April), *EOS* = *DOY* 340 (5 December), giving *LOS* = 242 days. The fitting results achieved *RMSE* (root mean square error, which reflects the mean magnitude of prediction errors) of 0.0080 and *R²* (coefficient of determination, which reflects the goodness of fit) of 0.7217, indicating that the double logistic model well captured the seasonal trajectory of the subtropical evergreen canopy and explained approximately 72% of the observed variance. This model exhibits a moderately favorable fit, though some bias persists. Combined with the trend analysis in Section 3.1 and the correlation analysis in Section 3.2, these findings indicate that *RGVI* is the most suitable index for delineating the canopy phenology of evergreen broad-leaved tree species.

**Figure 10:**
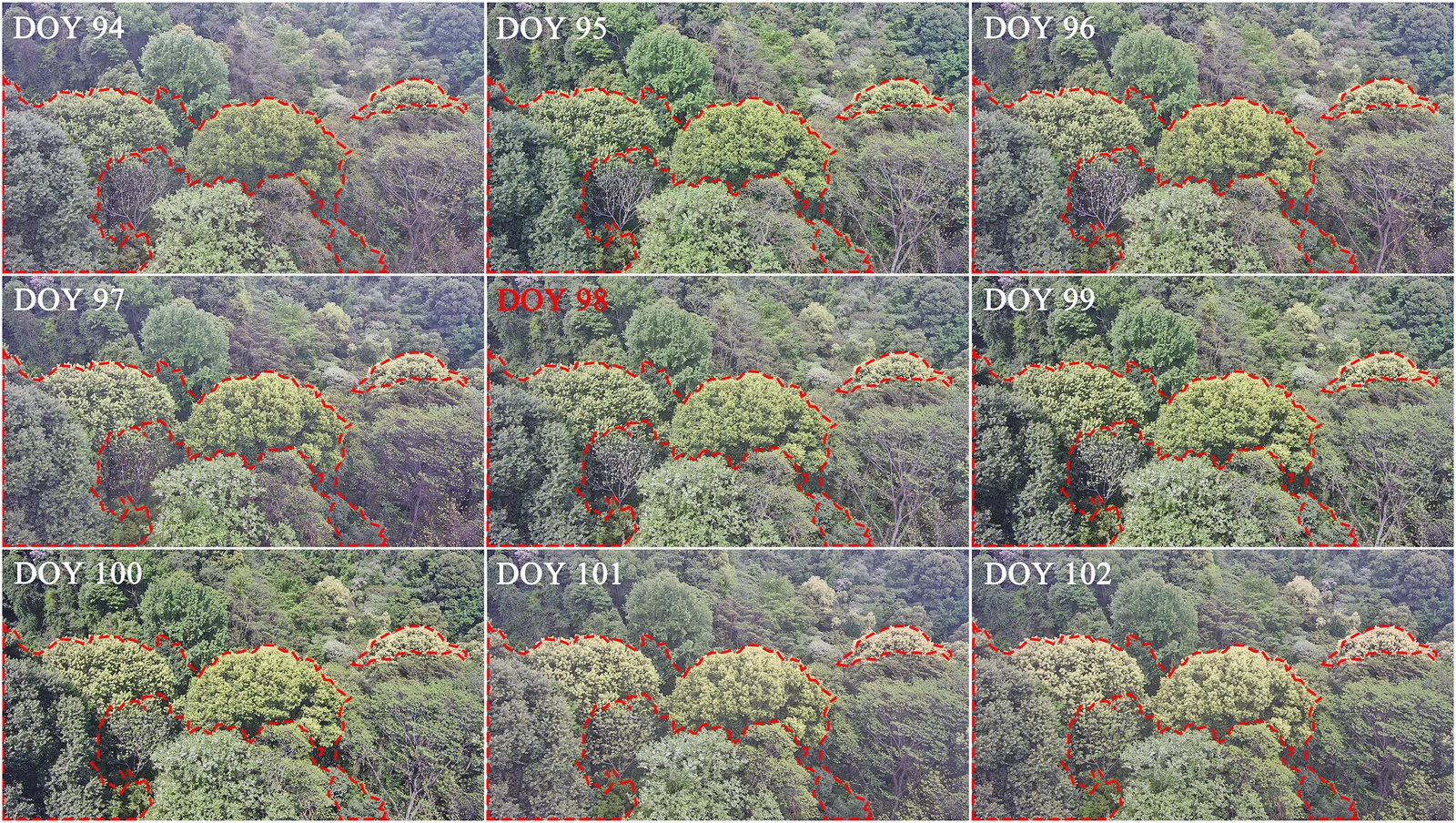
Phenological dynamics of *PhenoCam* observations before and after *SOS*.

**Figure 11:**
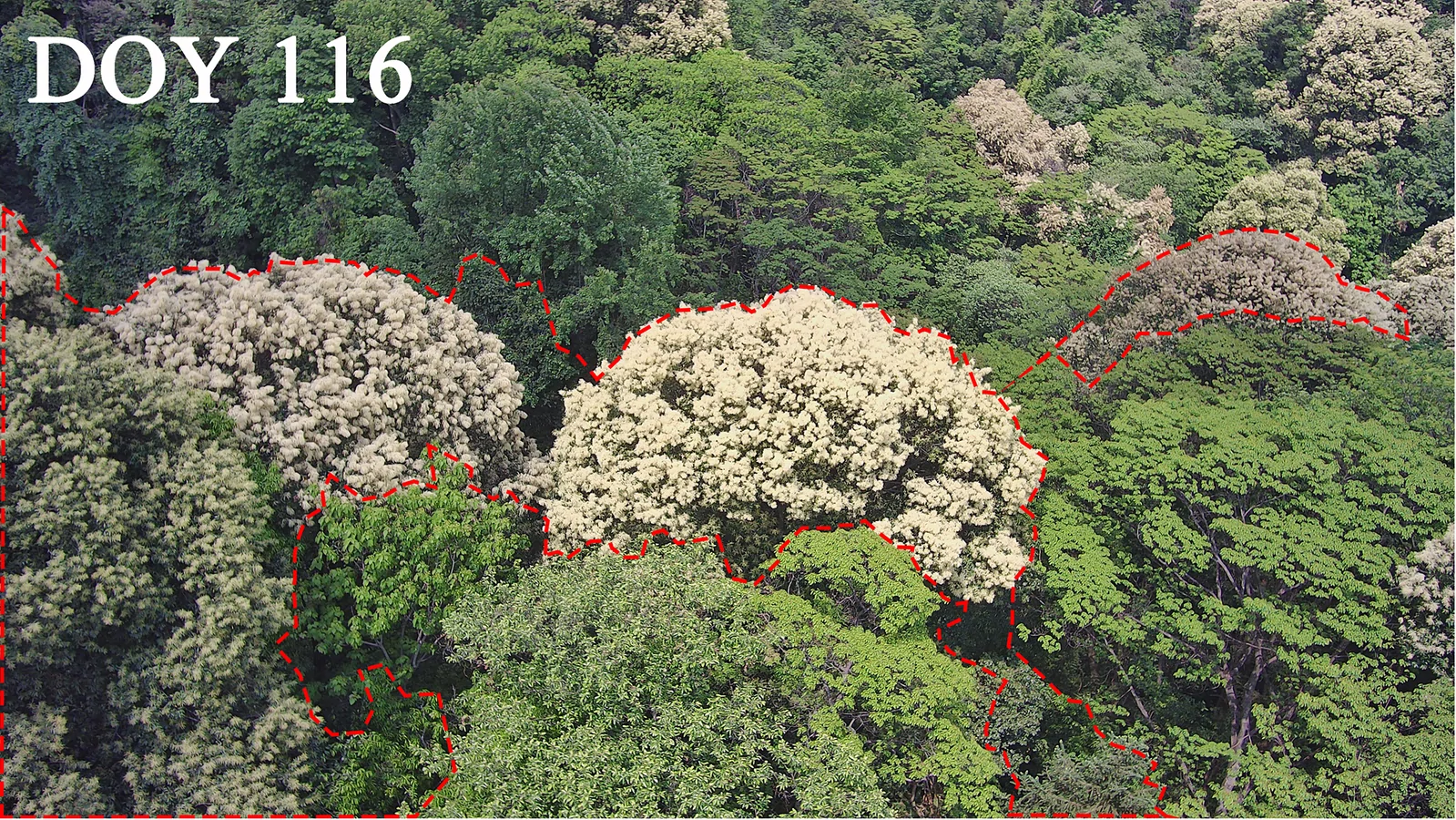
*PhenoCam*-observed image at peak flowering (*POP*, *DOY* 116).

**Figure 12:**
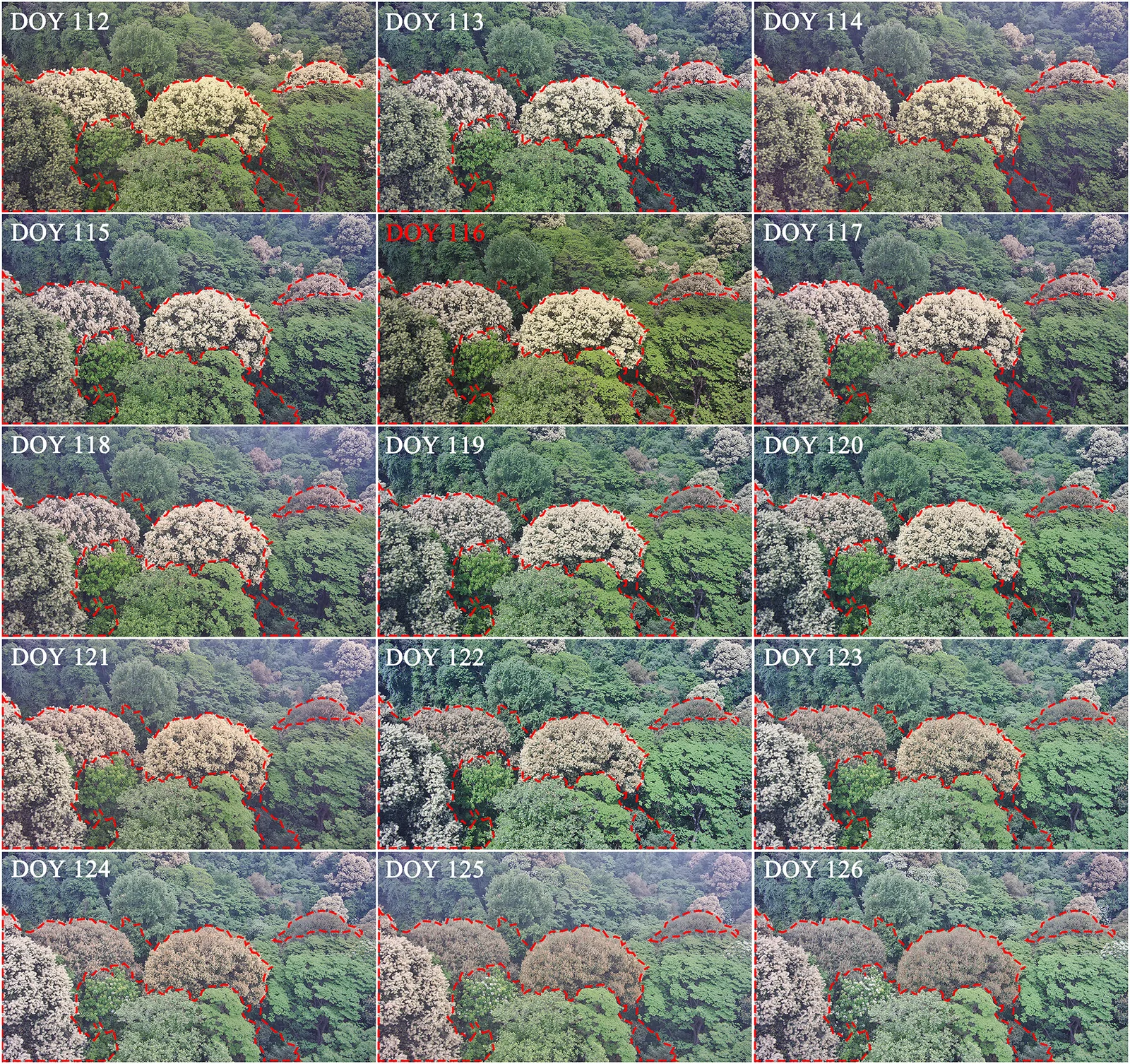
Phenological dynamics of *PhenoCam* observations before and after *POP*.

**Figure 13:**
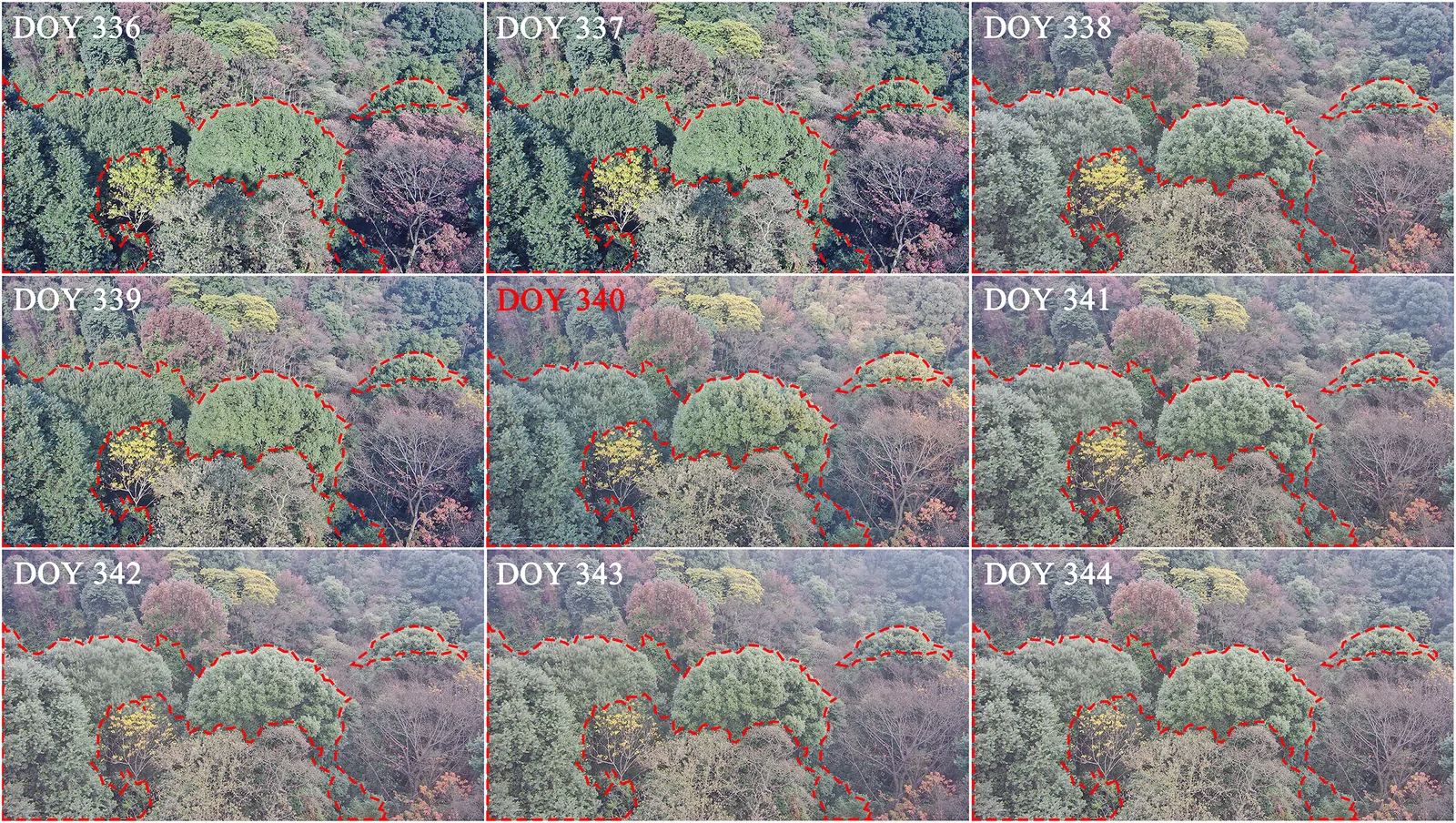
Phenological dynamics of *PhenoCam* observations before and after *EOS*.

To further validate the phenological timings derived from the *RGVI*-based double logistic model, canopy photographs were visually interpreted to accurately determine key phenological stages. The photograph-based observations indicated that *SOS* was characterized within a ±5-day window around its transition period, with a clear transformation of the onset of flowering beginning around *DOY* 98 (**Figure 10**). *POP* was defined over a broader period (*DOY* 112-126) to better capture the full flowering dynamics, during which canopy photographs showed a progressive increase in flowering intensity, reaching a peak around *DOY* 116 (**Figure 11**), followed by a gradual decline toward the end of flowering near *DOY* 126 (**Figure 12**). In contrast, *EOS* was also evaluated within a ±5-day window around its transition period (*DOY* 336-344). During this period, although no obvious changes were visually detectable in the evergreen broad-leaved trees’ canopy, phenological transition signals captured by *RGVI* may be driven by subtle structural and physiological variations at the leaf scale that are not directly observable in canopy images, as well as by changes in the surrounding deciduous broad-leaved trees, which showed an obvious deciduous change around *DOY* 340. It may influence the overall optical signal and contribute to the modeled *EOS* transition (**Figure 13**).

Overall, the phenological dates derived from *RGVI* fitting (*SOS* = *DOY* 98, *POP* = *DOY* 116, *EOS* = *DOY* 340) were highly consistent with the visually interpreted results from canopy images, showing only minor deviations within the corresponding temporal windows. In addition, combined with *PhenoCam* observations, the pronounced peak fluctuations around *SOS* and *POP* were attributed to the flowering in certain evergreen broad-leaved species, such as *Castanopsis fargesii*. This agreement between remotely sensed indices and *in situ* photographic observations further supports the reliability of *RGVI* for phenological characterization in subtropical evergreen broad-leaved forests.

### 3.4 Driving effect of environmental lag effect on key phenological events

Accounting for prior-season lags, the results show that different key phenological phases are significantly regulated by environmental factors from the preceding year (2023) and the study year (2024). The phenological metrics (*SOS*, *POP*, and *EOS*) used in this section were consistently derived from the *RGVI*-based double logistic time series in 2024. The *SOS* phase is mainly driven by temperature and spring light radiation conditions (*PAR* accumulates 712.45 mol·m^-2^), showing a sensitive response to cumulative temperature thresholds and dynamic cooling demand, as shown in **Table 4**, the spring *GDD* reaches 421.31 °C·d and the Dynamic *CP* accumulates 3.76 *CP*. The *POP* phase is more regulated by temperature stability (4.31 °C), radiation (Mean *PAR* is 118.54 μmol·m^-2^·s^-1^), and moisture conditions (precipitation accumulates 176.20 mm, and mean *SMC* reaches 38.73%), reflecting the dependence of the vigorous growth period on hydrothermal coupling conditions. The *EOS* phase is mainly controlled by mean air temperature (13.61 °C), radiation (Mean *PAR* is 72.96 *μ*mol·m^-2^·s^-1^) and accumulated high-temperature load (*CDD* reaches 92.53 °C·day), and is also affected by moisture conditions (precipitation accumulates 88.20 mm, and mean *SMC* reaches 36.82%), showing the comprehensive response of the autumn decline process to heat and moisture stress. In summary, when these conditions are met, canopy phenology in the study area will undergo a qualitative change in 2024.

**Table 4:** Driving factors of different key phenological events.

Key phenological events	Driving factors	Value	Unit
*SOS*	*GDD*	421.31	°C·day
	Dynamic *CP*	3.76	CP
	Spring *PAR*	712.45	mol·m^−2^
*POP*	*Temp* stability	4.31	°C
	Precipitation	176.20	mm
	Mean *SMC*	38.73	%
	Mean *PAR*	118.54	*μ*mol·m^−2^·s^−1^
*EOS*	*T_mean_*	13.61	°C
	*CDD*	92.53	°C·day
	Mean *PAR*	72.96	μmol·m^−2^·s^−1^
	Precipitation	88.20	mm
	Mean *SMC*	36.82	%

## 4 Discussion

### 4.1 Effects of environmental factors on canopy phenology of evergreen broad-leaved trees

This study showed that *NDVI*, *G_cc_*, *R_cc_* and *RGVI* had strong correlations with environmental factors of the current year (2024). Overall, radiation, temperature, moisture and air pressure were the most important influencing factors. This is consistent with the prevailing view that “water and heat conditions” are key influencing plant growth and phenological changes (Tang et al., 2023). *NDVI*, *R_cc_* and *RGVI* were significantly correlated with the “radiation-temperature” and “moisture-air pressure” environmental factors, whereas *G_cc_* showed only weak correlations with these factors and no correlation with “radiation-temperature.” This may be due to the following reasons. First, the monitored forests are mature, with canopy chlorophyll near saturation. Under this condition, *G_cc_* may be relatively insensitive to environmental variability. Second, the canopy is closed, with complex layering and heterogeneous light distribution. Consequently, light variations or temperature fluctuations within the selected *ROI* may affect photosynthesis primarily in lower strata. The chlorophyll content in the upper canopy remains comparatively stable, whereas the signals from lower layers are weaker and may not represent whole-canopy *G_cc_* dynamics. Therefore, the apparent effects of temperature and radiation are muted (Brown et al., 2017).

This study drew on previous popular research and selected several environmental lag effects that significantly impacted key phenological events (*SOS*, *POP* and *EOS*), such as accumulated temperature, cooling, accumulated rainfall, and accumulated radiation. The specific triggering conditions for the preceding year (2023) and the study year (2024) were also summarized. The results are generally consistent with research on hysteresis effects in the subtropics (He et al., 2023). However, the regional and temporal scope in the current study limited the ability to compare the environmental lag effects across spatial scales or to fit the relationship between these effects and phenological phases across timelines within the same region. Future research will accumulate more observational data from this station and conduct further analysis.

Currently, phenological and environmental studies on deciduous or evergreen coniferous species are more extensive (Delpierre et al., 2016), while relevant research on evergreen broad-leaved species has not yet become systematic. Therefore, broader research is needed to study how climate change specifically affects the phenological changes of evergreen broad-leaved species.

### 4.2 Canopy latent phenological changes and phenological phase extraction

The phase extraction and analysis conducted in this study revealed inconsistent intra-annual trends among latent phenological indicators for evergreen broad-leaved canopies in the study area in 2024. Despite prior work using *NDVI* and *G_cc_* as primary phenology indicators (Cui et al., 2025) and treating *R_cc_* and *RGVI* as mainly autumn- and winter-sensitive (Chen et al., 2025; Ide and Oguma, 2010), results here showed *NDVI* and *G_cc_* underperforming *R_cc_* and especially *RGVI* for phenological prediction and phase extraction, diverging from earlier phase-extraction studies (Aasen et al., 2020; Zhang et al., 2024). In contrast, *RGVI* exhibited greater stability and sensitivity across phenological stages and was therefore identified as the most robust metric and ultimately selected for subsequent phenological analyses in this study. The extracted results generally aligned with expectations for subtropical evergreen broad-leaved forests, but *EOS* was estimated more accurately than *SOS* (Ren et al., 2021). These findings indicate *RGVI* is not limited to capturing autumn and winter variations in evergreen coniferous species; instead, it plays a key role in evergreen canopy phenology.

The study area has a subtropical humid monsoon climate and evergreen broad-leaved trees continually renew foliage, maintaining an apparently permanent “green” canopy. Consequently, *NDVI* associated with chlorophyll absorption in the red band and strong near-infrared reflectance from cellular structure (Huete et al., 2002), and *G_cc_* also related to chlorophyll (Keenan et al., 2014), exhibit weak seasonality, fluctuating around a stable level. By contrast, *R_cc_*, which is associated with anthocyanins and carotenoids (Li et al., 2023b; Liu et al., 2020), shows pronounced seasonal variations, with higher values in summer - autumn and lower values in winter - spring. Interpreting the indices functionally, *NDVI* reflects vegetation growth, *G_cc_* tracks photosynthetic greenness, and *R_cc_* is sensitive to pigment dynamics and chlorophyll decline. In evergreen broad-leaved forests, chlorophyll tends to be comparatively stable, whereas anthocyanins and carotenoids fluctuate. Building on these pigment-based responses, *RGVI* integrates variations in both chlorophyll-related greenness and accessory pigments (e.g., anthocyanins and carotenoids), thereby capturing more comprehensive canopy biochemical dynamics across seasons. Nonetheless, further vegetation physiological experiments are needed to clarify how pigment dynamics drive phenological changes. Prior work has shown that *PhenoCam*-derived *R_cc_* is a sensitive indicator of canopy phenology in evergreen biomes where seasonal greenness changes are subtle. As such, *R_cc_* often outperforms *G_cc_* in detecting transitions and in providing a stable and continuous signal of physiologically driven “cryptic phenology” (Kumar et al., 2025; Liu et al., 2020; Toomey et al., 2015). However, the dynamic range of the time series of *R_cc_* is relatively small. Given that *RGVI* is a derivative index integrating pigment-related information, it is expected to provide an even stronger response to cryptic phenology dynamics. Nevertheless, although *RGVI* has demonstrated robust performance in phenological extraction, its underlying physiological and biochemical interpretation warrants further investigation.

Perhaps this study represents a new attempt to investigate cryptic phenology in evergreen broad-leaved forests. Furthermore, the results underscore that near-surface *PhenoCam* observations, owing to their proximity and high temporal/spatial resolution, offer clear advantages over satellite monitoring for fine-scale and detailed canopy phenology studies (Aasen et al., 2020), and have promising prospects in future research in evergreen broad-leaved systems. *PhenoCam*s observations are, however, susceptible to external influences, including illumination and rainfall variability, occasional lens occlusion, and camera resolution effect, all of which can introduce noise (Khare et al., 2021; Lu et al., 2022). Thus, systematic data screening and larger monitoring datasets are required to further validate the ability of *RGVI* to characterize subtropical evergreen canopy phenology. Integrating manual field observations with enhanced modelling approaches should also improve the delineation of phenophases such as flowering and fruiting in evergreen broad-leaved species, and field observations in this study further indicate that flowering is a key driver of the observed phenological variations.

**Figure 14:**
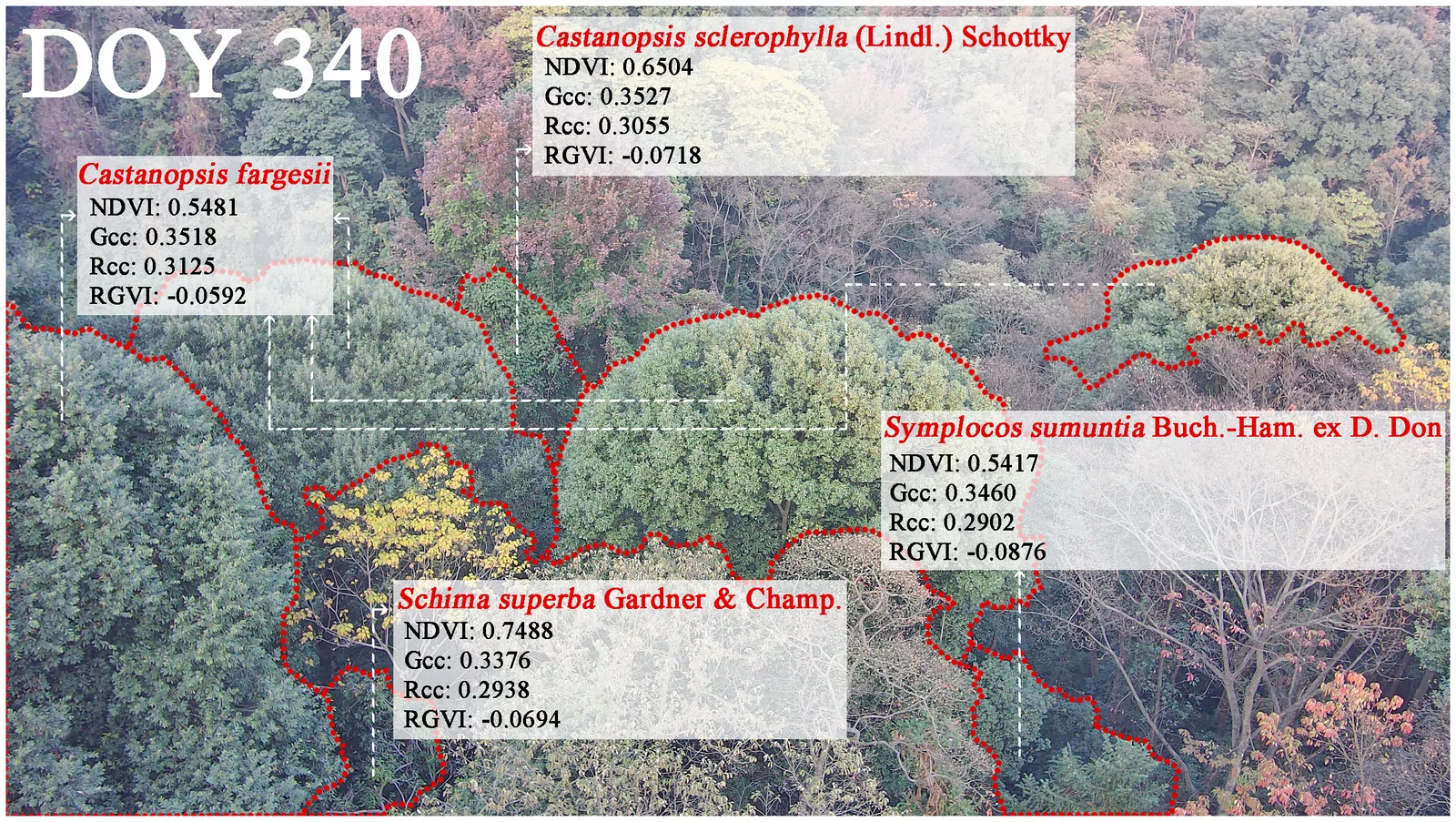
*NDVI*, *G_cc_*, *R_cc_*, and *RGVI* extracted from *PhenoCam* images for individual evergreen broad-leaved trees (*DOY* 340, 2024, as an example).

**Figure 15:**
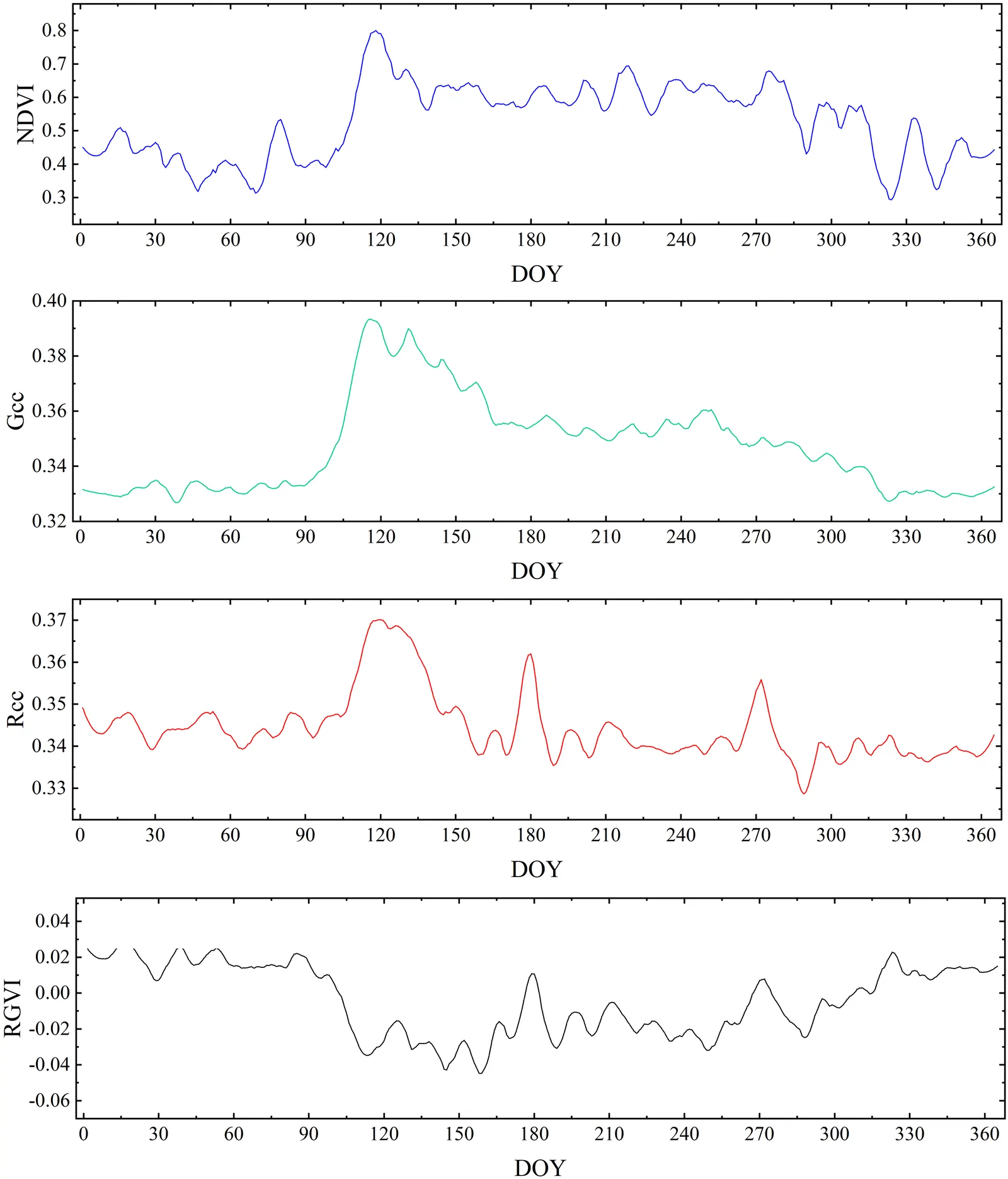
*NDVI*, *G_cc_*, *R_cc_* and *RGVI* of deciduous broad−leaved trees derived from *PhenoCam* observations throughout 2024.

This study focused on a single sample site (Jiangxi Dagangshan National Field Station) and a single year. Future work should incorporate multiple monitoring stations and longer time series to validate cryptic-phenology patterns across spatial and temporal scales (Richardson et al., 2018). In addition, complementary measurements of other plant organs, including the trunk (Silvestro et al., 2025) and the root system (Malyshev et al., 2023), are needed to fully resolve concealed phenological processes in trees (Delpierre et al., 2016). On this basis, we will also plan to conduct an in-depth observation of the phenological changes of individual tree species in evergreen broad-leaved forests. As illustrated in **Figure 14**, individual tree crowns of different species (*Castanopsis fargesii*, *Castanopsis sclerophylla* (Lindl.) Schottky, *Symplocos sumuntia* Buch.-Ham. ex D. Don, *Schima superba* Gardner & Champ.) can be clearly delineated from *PhenoCam* imagery, allowing for the extraction of *NDVI* and RGB-based chromatic indices (*G_cc_*, *R_cc_*, and *RGVI*) at the single-tree level. Our future work will also further extend this framework to deciduous species and conduct comparative analyses between deciduous and evergreen broad-leaved forests, with **Figure 15** illustrating the phenological dynamics of deciduous trees observed by *PhenoCam* in 2024.

However, the present study primarily focuses on community-level phenological dynamics of evergreen broad-leaved forests. Moreover, analyses at the individual-tree scale are computationally intensive and require more refined crown segmentation and time-series processing. Therefore, detailed investigations at the individual-tree level will be further explored in future studies.

## 5 Conclusions

*In situ PhenoCam* observations from the Dagangshan National Field Station were carried out in 2024 to characterize the canopy cryptic phenology of subtropical evergreen broad-leaved forests, together with environmental variables from 2023 and 2024 to account for potential lagged and contemporaneous effects of environmental drivers. The main results and conclusions are as follows:

1. Among latent phenological indicators, *NDVI* and *G_cc_* varied slightly, while *R_cc_* and *RGVI* showed the strongest alignment with intra-annual climatic variations.
2. Radiation, temperature, moisture and air pressure were identified as the primary environmental factors associated with canopy phenology in the study area.
3. *RGVI* and *R_cc_* was positively correlated with all environmental factors. *NDVI* correlated positively with “radiation–temperature” but negatively with “moisture–air pressure”. In contrast, *G_cc_* showed weaker or non-significant correlations, plausibly reflecting external influences and the structural complexity of a closed, multilayered canopy.
4. Leveraging latent phenological indicators, *RGVI* proved effective for simulating vegetation growth dynamics in evergreen broad-leaved canopies by fitting a double Logistic model, outperforming *NDVI*, *G_cc_* and *R_cc_* for phenophase detection under year-round green conditions. It was revealed that the growing season commenced in early April (*DOY* 98), peaked by late April (*DOY* 116) and commenced a significant decline in early December (*DOY* 340), spanning a growing-season length of 242 days.
5. Phase-dependent lag effects were evident. Spring onset was governed by cumulative temperature and radiation; peak growth by hydrothermal coupling and temperature stability: and autumn senescence by accumulated thermal exposure under interacting radiation and moisture effects.
6. The findings support the use of *RGVI*-based phenology for monitoring, modelling and forecasting in subtropical evergreen forests, providing a theoretical basis for future studies at finer spatial and temporal scales.

Future work will expand to multi-site, multi-year observations, integrate more detailed manual phenophase records (e.g. flowering, fruiting) with improved modelling, and incorporate physiological measurements to clarify pigment-phenology linkages in evergreen systems.

## Data Availability

The datasets presented in this study can be found in online repositories. The names of the repository/repositories and accession number(s) can be found below: Zenodo, https://doi.org/10.5281/zenodo.20328126.
